# Determining the Role of Acicular Ferrite Carbides in Cleavage Fracture Crack Initiation for Two Medium Carbon Microalloyed Steels

**DOI:** 10.3390/ma16227192

**Published:** 2023-11-16

**Authors:** Gvozden Jovanović, Dragomir Glišić, Stefan Dikić, Bojan Međo, Branislav Marković, Nikola Vuković, Nenad Radović

**Affiliations:** 1 Institute for Technology of Nuclear and Other Mineral Raw Materials, Metallurgical and Environmental Engineering, Bulevar Franše d’Eperea 86, 11000 Belgrade, Serbia; b.markovic@itnms.ac.rs (B.M.); n.vukovic@itnms.ac.rs (N.V.); 2 Faculty of Technology and Metallurgy, University of Belgrade, Karnegijeva 4, 11120 Belgrade, Serbia; gile@tmf.bg.ac.rs (D.G.); bmedjo@tmf.bg.ac.rs (B.M.); nenrad@tmf.bg.ac.rs (N.R.)

**Keywords:** cleavage fracture, forging steel, finite element analysis, acicular ferrite, MIPAR, ABAQUS

## Abstract

Two medium-carbon microalloyed steels with a predominant acicular ferrite microstructure were investigated in this study in order to determine the initial micro-crack formation mechanism and the role of acicular ferrite structure in cleavage fracture. In order to ensure cleavage fracture, samples were investigated at −196 °C for uniaxial tension and four point bending fracture. Previous investigations have shown that cleavage fracture for steels with a predominant acicular ferrite microstructure has not been initiated by the fracture of coarse TiN particles as in ferrite-pearlite, bainite, or martensitic microalloyed steels. The average maximal thickness of cementite plates measured in this work is 0.798 µm and 0.966 µm, for V and TiV steel, respectively. The corresponding stress values required for their fracture according to Griffith’s equation are 1970 MPa and 1791 MPa, respectively. Estimated values of the effective surface energy for the V steel with an average cementite volume fraction of 3.8% range from 40 Jm^−2^ to 86 Jm^−2^, and for the TiV steel with an average cementite volume fraction of 18.3% range from 55 Jm^−2^ to 82 Jm^−2^. The fracture of coarse cementite plates was found to not to be responsible for the cleavage fracture initiation in case of both steels.

## 1. Introduction

Previous studies over the last two decades have found that acicular ferrite (AF) in the microstructure improves the toughness of low-carbon and welded pipeline steels [1,2,3,4,5,6,7]. By continuous cooling from above Ac1 temperatures, a range of heterogeneous microstructures can be formed, depending on the cooling rates and the chemical composition; thus, AF was first noticed in the heat-affected zones (HAZ) of welded steel [8]. The microstructure of medium-carbon microalloyed steels can consist of ferrite, perlite, bainite, acicular ferrite, martensite, and retained austenite [9,10]. Particularly, AF is generated by the bainitic reaction; however, unlike bainite, which develops from the austenite grain boundary, AF nucleates intragranularly. Since AF nucleates intragranularly at inclusions, generating fine interlocking laths and/or plates, it enhances toughness presumably by causing propagating cracks to deflect at high-angle borders between ferrite laths and plates [11,12,13].

Continuously cooled medium-carbon microalloyed steel samples from the temperature of austenitization or hot working also exhibit the formation of AF alongside other microconstituents, principally ferrite and pearlite [10,14]. Promising results in increasing the toughness of medium-carbon microalloyed steels with a primarily acicular ferrite structure have been observed [1,15,16,17]. However, when it comes to continuously cooled medium carbon microalloyed steels, fracture studies are primarily focused on ferrite-pearlite and bainite structures [18,19]. Various advancements have been made regarding crack propagation in different fields using FEM modeling [20,21]. Furthermore, when it comes to modeling fracture mechanics, there have been comprehensive studies of ferrite-perlite microstructures due to their simplicity [22,23,24,25], but for crack initiation, some of those mechanics can be redesigned for the AF matrix.

Studying cleavage fracture initiation and propagation of steel with an AF matrix has been mostly done on isothermally heat-treated steel samples that have a controlled and well-defined structure [8,15,26]. However, the cleavage fracture in continuously cooled medium-carbon microalloyed steels with a mainly acicular ferrite structure has received little attention. Previous investigations have shown that cleavage fracture in medium carbon microalloyed steels with a predominant AF microstructure has not been initiated by the fracture of coarse particles such as TiN, as is the case for steels with ferrite-pearlite, bainite, or martensitic microstructures [27]. Furthermore, the influence of the AF on cleavage initiation mechanisms has been associated with the fracture of ferrite-pearlite aggregates (grains with the same crystallographic orientation) or coarse carbides in ferrite under high plastic strains near the notch of the four-point bending specimen [28,29]. This work represents a continuation of these investigations in order to determine the initial micro-crack formation mechanism, establish the critical stage of the cleavage fracture for such a mechanism, and further clarify the role of AF structure in cleavage fracture for continuously cooled medium carbon microalloyed steels.

## 2. Materials and Methods

The chemical compositions of two commercial medium-carbon microalloyed steels are given in Table 1. The V and TiV microalloyed steels were received as hot-rolled rods, 19 mm and 22 mm in diameter, respectively. The as-received rods were homogenized at 1250 °C for 4 h in an argon atmosphere, followed by oil quenching in order to eliminate rolling texture. Afterward, the specimens were reaustenitized at 1250 °C for 30 min in an Argon atmosphere and then cooled in still air.

Metallographic specimens were cut from the rods in a transverse direction and mechanically prepared by grinding and polishing. In order to distinguish cementite particles, samples were first etched in a 2% picric acid-ethanol solution [30]. The microstructure of the steels was examined by light microscopy using a MeF3 microscope (Reichert-Jung, Vienna, Austria) equipped with a “Leica” digital camera (Leica Microsystems, Wetzlar, Germany) and digital image acquisition software.

### 2.1. Image Analysis

Quantitative analysis of the microstructures was performed using MIPAR™ software (v4.3.0) [31]. Due to the repetitiveness of the AF microstructure for measuring the thickness of the cementite laths between the ferrite needles, 20 representative sample pictures were analyzed using the MIPAR software for both steels using different custom-made recipes. However, for calculating the area fraction of the three phases of interest (cementite, ferrite, and perlite), 200 micrographs were analyzed in order to get a proper understanding of the distribution of the measured cementite phase in the investigated microstructure.

### 2.2. Finite Element Analysis 

**Figure 1:**
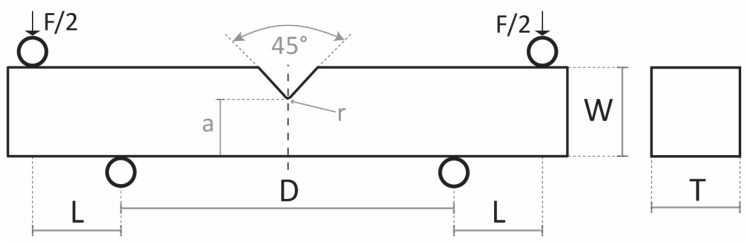
Schematic of a Griffiths–Owen 4PB specimen with respective measurements used for modeling: T = 12.7 mm, W = 12.7 mm, a = 8.47 mm, D = 38.1 mm, L = 12.7 mm, r = 0.25 mm.

Tests were carried out at the temperature of liquid nitrogen (−196 °C) with a constant crosshead speed of 0.1 mm min^−1^ in order to investigate the cleavage fracture by four-point bending (4PB). Four-point bending notched specimens are the same type as those used by Griffiths–Owen, illustrated in Figure 1 [32].

**Figure 2:**
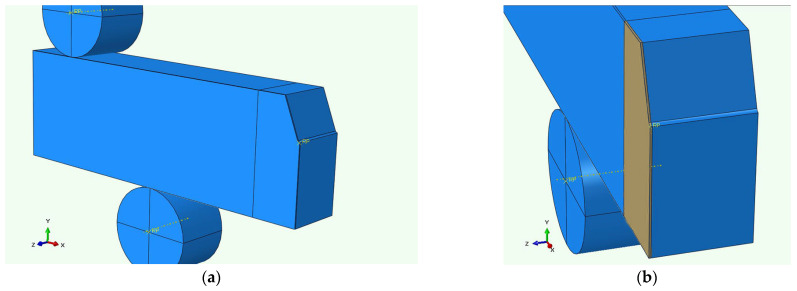
ABAQUS FEA global model assembly of the 4PB specimen (**a**) with shaded volume of the sub-model (**b**).

In order to calculate stresses and strains in the four-point bending specimens the finite element analysis (FEA) was performed using ABAQUS software (v6.12) [33]. One quarter of the specimen was modeled in three dimensions as a global model, as presented in Figure 2a.

Mechanical properties were modeled by the true stress-strain curves created using polynomial regression of the experimental data obtained from the uniaxial tension testing at −196 °C. Tensile testing was performed in a liquid nitrogen bath, using cylindrical specimens 6 mm in diameter and 30 mm long (EN ISO 6892-1) [x] at a constant crosshead speed of 0.1 mm min^−1^, giving an initial strain rate of the same order of magnitude (10^−5^ s^−1^) as the four-point bending test. Elastic modulus and Poisson’s ratio of 200 GPa and 0.28, respectively [32], were used to simulate the steels’ elastic response.

The model was set up by assigning the displacement of the Reference Point in the center of the upper pin while the lower one was immovable. The reaction force at the upper pin corresponded to the same experimentally determined fracture load. Experimental values of the fracture load, F_max_, nominal bending stress, σ_f_, along with corresponding modeled displacements, U2, are presented in Table 2.

**Figure 3:**
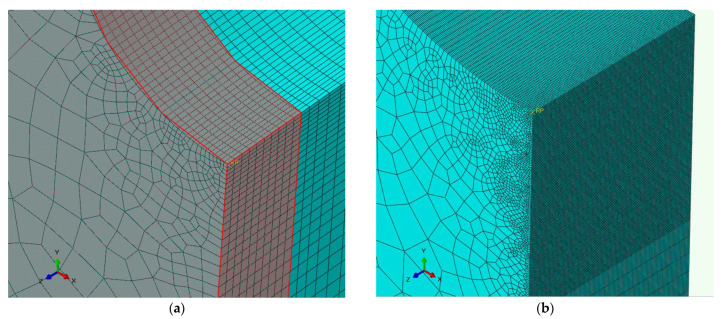
FEA meshing of the specimen edge: (**a**) global model; (**b**) sub-model (the sub-model area is shaded red in the global model).

In order to acquire a more accurate distribution of the stress and strain, a sub-model was employed for a portion in the middle of the 4PB sample model, as shown in Figure 2b. The sub-model was 0.127 mm wide, and its element size is 0.001 mm along the notch edge (along the z axis) as well as 0.127 mm from the notch root (along the y axis). The difference between the element sizes of the global model and sub-model is clearly demonstrated in Figure 3. In both cases, a hexagonal eight-node element type with reduced integration (C3D8R) was used, with geometric nonlinearity taken into account.

### 2.3. Cleavage Fracture Calculation

A JEOL JSM-7001F Field Emission Scanning Electron Microscope (Jeol, Tokyo, Japan) coupled with Xplore 15 Energy-dispersive X-ray spectrometer (Oxford Instruments, Abingdon, UK) was used for fracture investigation. The origins of fracture were discovered by following traces on the fracture surfaces and cleavage facets. From the SEM micrographs, the distance of the fracture initiation point from the notch root, X_0_, and the size of the first cleavage facet, were measured. An ellipse with main axes matching the facet’s maximum and minimum ferret diameters (D_max_ and D_min_, respectively) was used to estimate the initial cleavage facets’ sizes.

The FEA estimated maximum principalstress distribution at the distance of the cleavage initiation point from the notch root, X_0_, was used to calculate the local cleavage fracture stress, σ_F_* [18,19]. Griffith’s equation connects critical cleavage fracture stress, σ_F_*, and the initial cleavage facet dimensions:

(1) $$\sigma_{F}^{*}=\sqrt{\frac{\pi \cdot E\cdot \gamma }{\left({1-\nu }^{2}\right)\cdot D_{eff}}}$$

where γ is the effective surface energy, D_eff_ is the effective diameter of the first cleavage facet, E is the modulus of elasticity, and ν is the Poisson’s coefficient. The effective diameter, D_eff_, was calculated by the following formulas [17,18]:

(2) $$D_{eff}=\frac{D_{min}}{\phi^{2}}\frac{\pi^{2}}{4}\Rightarrow \phi =\frac{3\pi }{8}+\frac{\pi }{8}{\left(\frac{D_{min}}{D_{max}}\right)}^{2}$$

The effective surface energy from Equation (1), γ, was determined from the plot of the local critical cleavage fracture stress, σ_F_*, versus the reciprocal square root of the first cleavage facet effective diameter, D_eff_^−1/2^. The slope of the plot, k, is then used to calculate the effective surface energy by the following equation:

(3) $$\gamma =\frac{1-\nu^{2}}{E\cdot \pi }\cdot k^{2}$$

## 3. Results

**Figure 4:**
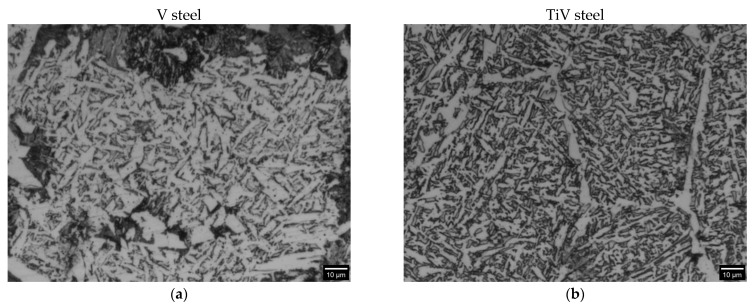

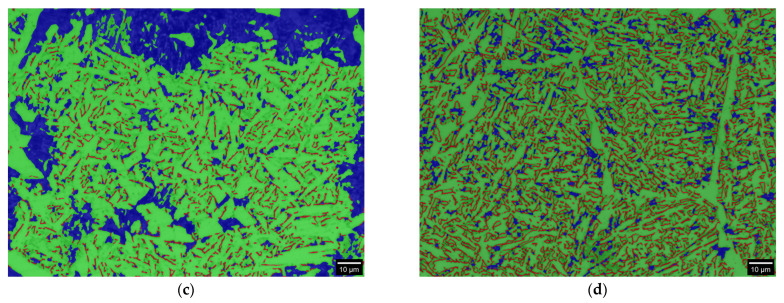
Microstructure and phase distribution of both V and TiV steels given by MIPAR software (the overlay is at 50% opacity Red = Cementite; Green = Ferrite; Blue = Perlite) Grayscaled images (**a**,**b**); RGB modeled overlay (**c**,**d**).

Micrographs of the two medium carbon steels microalloyed with V and TiV in Figure 4 show that the microstructure in both steels generally consists of three components: ferrite, pearlite, and acicular ferrite, with the latter being the most prevalent. Comparing the microstructures of continuously cooled V and TiV steels, it is evident that the V steel has a noticeable higher content of pearlite. The results of the quantitative analysis of the microstructures are given in Table 3, together with the statistical representation of all measurements. For the purpose of the quantitative analysis, microconstituents were divided into three simple segments: ferrite, which, beside grain boundary ferrites and intragranullar ferrite grains, encompasses acicular ferrite plates, then perlite nodules, and finally individual cementite plates located between the acicular ferrite plates. Alongside the micrographs in Figure 4a,b, a digital phase overlay taken for the analysis is also given in Figure 4c,d.

Taking the values of the mean area fraction of the microconstituents as the average volume fraction (Table 3), it confirms that TiV steel has a considerably lower volume fraction of pearlite and a higher volume fraction of cementite plates than V steel, which is reasonable since TiV steel contains a higher amount of acicular ferrite in the structure. In terms of the average volume fraction of cementite, TiV steel exhibits a significantly higher value of 18.3% than V steel with only 3.8% (Table 3).

Segmentation of acicular ferrite from the other forms of ferrites is a difficult task because the size and shape of the acicular ferrite plates and small intragranular or grain boundary ferrite grains in the plane of the image could be very similar. Namely, acicular ferrite in continuously cooled steels can be described as a mixture of the lower and upper acicular ferrite isothermal morphologies [34]. Particularly lower acicular ferrite is depicted by sheaves of nearly parallel plates or laths with similar crystallographic orientation, in contrast to upper acicular ferrite, which is characterized by the structure of fine interlocking ferrite plates with high crystallographic misorientation [1,28,35,36]. It follows that coarser acicular ferrite plates in continuously cooled microstructures are often difficult to distinguish from the small proeutectoid ferrite grains.

**Figure 5:**
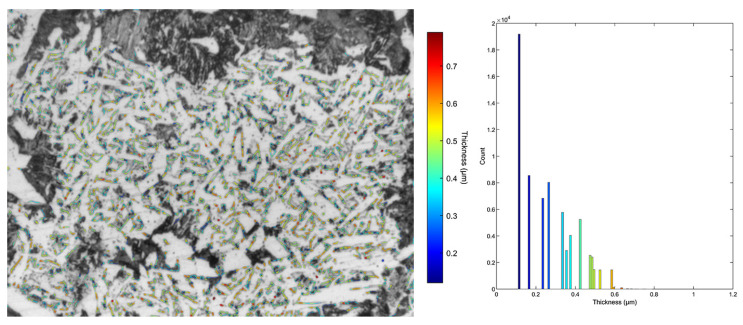
MIPAR local thickness measurement of cementite with corresponding scale and histogram for representative V optical micrograph.

**Figure 6:**
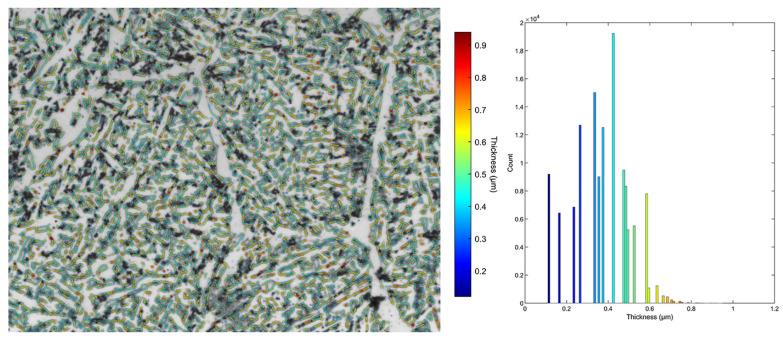
MIPAR local thickness measurement of cementite with corresponding scale and histogram for representative TiV optical micrograph.

To clarify the role of cementite particles in cleavage fracture initiation, the thickness distribution of the cementite plates within the acicular ferrite was also assessed from the light microscopy micrographs. The statistical representation of cementite phase thickness measurements for each investigated steel is summarized in Table 4. Figure 5 and Figure 6 show examples of the cementite phase segmentation on micrographs, together with the related cementite plate thickness distribution.

Comparing the two steels, it is evident from Table 4 that, on average, cementite plates in TiV steel are thicker than in V steel. Cementite plates in TiV steel tend to have a slightly higher average mean local thickness (0.374 μm) compared to V steel (0.264 μm). The average maximum local thickness in TiV steel (0.966 μm) is somewhat higher than in V steel (0.798 μm). Comparing the histograms of local thickness (Figure 5 and Figure 6) for the two steels, it can be clearly seen that the highest count for the V steels is for the minimum thickness (0.118 μm), while for the TiV steel, the highest count is for the median thickness (0.424 μm). Furthermore, from Table 4, the minimum thickness is approximately the same for both steels across all sampled micrographs. This is most likely a consequence of the light microscopy limitations, as cementite plates with the lowest thickness cannot be observed under the light microscope at the magnification used. However, from the point of view of fracture analysis, larger particles are of interest, considering their role in cleavage fracture initiation [37,38].

It is well accepted that AF forms by the same reaction as bainite, except that nucleation takes place in the austenite grain interiors, at the second-phase particles [39,40]. In the upper bainite, cementite particles are distributed between the ferrite plates, while the interior of the bainite ferrite plates itself is free of carbides [41]. Lower bainite is characterized by sheaves of ferrite plates with small cementite particles precipitating in their interiors. This classification is based on isothermally formed morphologies of upper and lower bainite as well as upper and lower AF. Since it could be assumed that continuously cooled bainite or AF consists of a mixture of both morphologies, the relatively coarse cementite plates measured in this work could be associated with the upper AF morphology.

**Figure 7:**
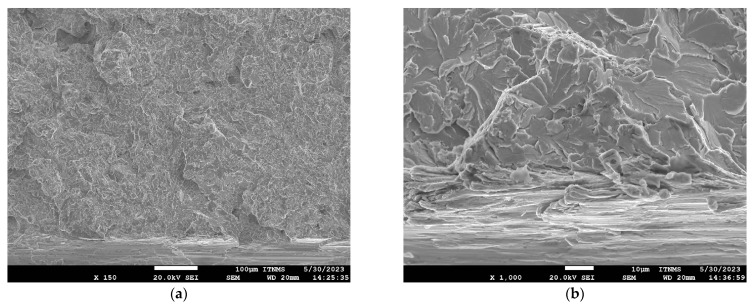

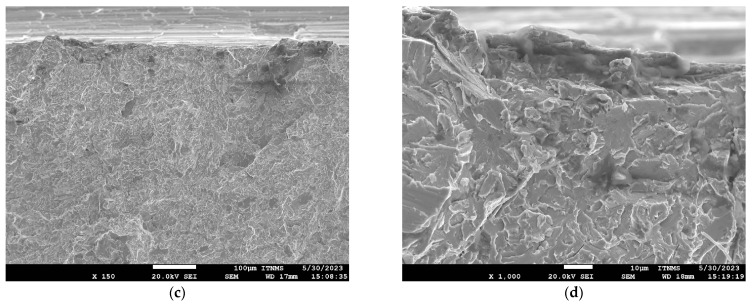
Mirrored surfaces of 4PB cleavage fracture with the same potential source for V02 steel sample. Left fracture surface (**a**,**b**); Right fracture surface (**c**,**d**).

**Figure 8:**
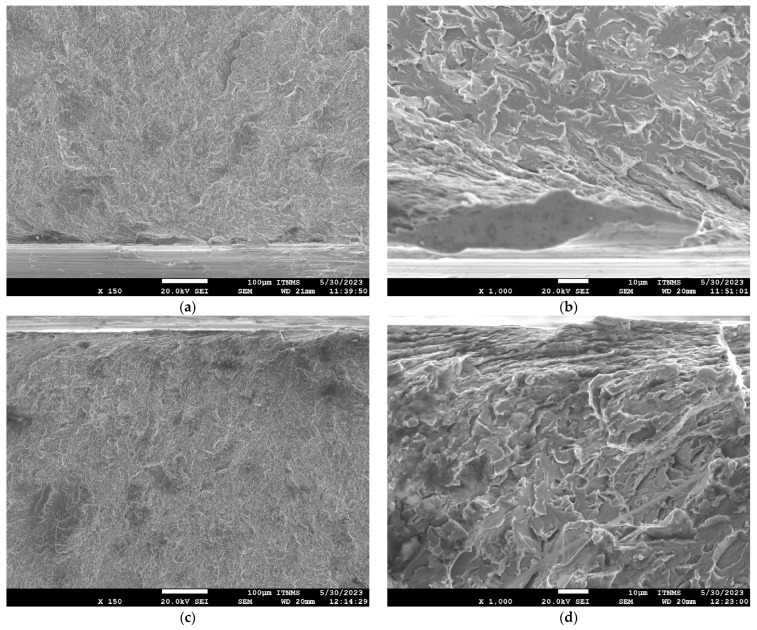
Mirrored surfaces of 4PB cleavage fracture with the same potential source for TiV-02 steel sample. Left fracture surface (**a**,**b**); Right fracture surface (**c**,**d**).

SEM fractographs for V and TiV steel in Figure 7 and Figure 8, respectively, represent two matching halves of the fractured 4PB specimens at different magnifications. Chevron lines at the fracture surface observable at low magnification in Figure 7a,c and Figure 8a,c point at the area near the notch root. SEM fractographs at high magnifications in Figure 7b,d and Figure 8b,d show small cleavage facets of irregular shapes with traces that can be characterized more as feather markings than river lines. Feather markings are fan-shaped cleavage steps that spread in the direction of the crack propagation [42,43] and therefore can be traced back to the origin of the fracture. In this case, for both V and TiV steel samples, the origin of the cleavage fracture is almost at the notch root of the 4PB specimen (Figure 7b,d and Figure 8b,d). The edge at the notch root of the 4PB specimen is visibly deformed. There are no broken second-phase particles at the fracture origin. Small irregular surfaces of the cleavage facets with very fine cleavage steps make the search for the fracture initiation site difficult, and in some cases, multiple fracture origins were taken into consideration. That is the reason why in some samples, multiple cleavage facets as potential sites of cleavage initiation were examined, as shown in Table 5. The shape and size of the cleavage facets can be related to fractured microconstituents: polygonal ferrite grains and pearlite nodules that give larger facets, or acicular ferrite plates or sheaves of plates, depending on the type of AF, which give small irregularly shaped facets with many tear lines along the edges (Figure 7 and Figure 8). Furthermore, groups of irregular facets without clear boundaries can be related to plates or grains with similar crystallographic orientation. Larger facets may also be interpreted as aggregates of ferrite and pearlite grains with the same crystallographic orientation that can be observed in micrographs in Figure 4, Figure 5 and Figure 6 as relatively coarse proeutectoid ferrite grains neighboring the perlite nodules.

**Figure 9:**
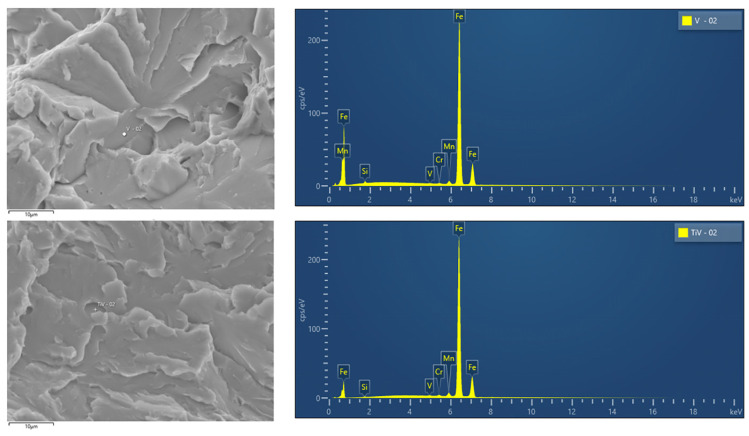
EDS spectrum for potential source of fracture for V-02 and TiV-02 steel sample.

EDS analysis of the small flat surfaces at the apex of the feather lines at the cleavage origin in V and TiV steel samples shown in Figure 9 confirms that there are no second-phase particles, such as TiN or complex MnS-based inclusions. Peaks in the EDS spectrum for Fe are distinct among the usually present alloying elements, Mn, Si, V, and Cr, in both examined steels, which clearly indicates that the facets at the detected sites of the fracture initiation do not belong to any broken second-phase particle. Therefore, it could be concluded that the initial microcrack in the cleavage fracture process was not formed by the fracture of a coarse second-phase particle. Cleavage fracture in medium-carbon microalloyed steels with ferrite-pearlite, bainite, or martensite structures is generally initiated by the fracture of a coarse second-phase particle. In the vast majority of cases, broken coarse TiN particles (usually 2–6 μm in diameter) in the zone of peak values of the maximum principal stress in front of the notch of the 4PB specimen are responsible for the cleavage fracture initiation [18,27,32,33,34,35,43,44,45]. Conversely, there are certain previous studies that have reported cases where coarse TiN particles have remained inactive in the cleavage fracture initiation process in microalloyed steels with fine acicular ferrite structures [28,29]. In the present work, no broken second-phase particles were detected at the cleavage fracture origin in any of the samples in either V or TiV steel.

**Figure 10:**
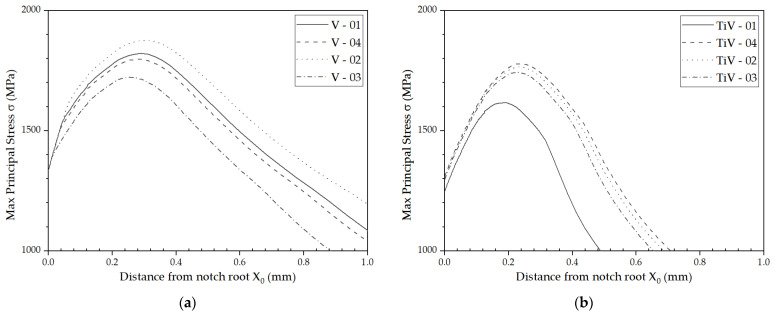

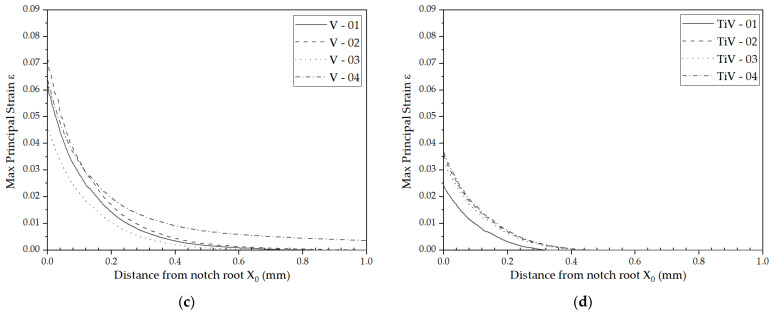
Max principal stress σ and strain ε distribution at the fracture along the notch root. V steel samples (**a**) stress and (**c**) strain; TiV steel samples (**b**) stress and (**d**) strain.

The position of the fracture origins along the distance from the notch root, X_0_, and the dimensions of the first cleavage facets, measured as maximal and minimal facet diameters, D_max_ and D_min_, are summarized in Table 5. The values of the distances from the notch root to the cleavage initiation sites, X_0_, for V steel samples are between 12 and 54 μm and from 41 to 131 μm for the TiV steel samples. The values of the effective cleavage facet diameter, D_eff_, calculated from the maximal and minimal facet diameters, D_max_ and D_min_, also summarized in Table 5, are somewhat higher for TiV steel. Although the differences can be considered negligible by taking into account the standard error of the measurements, since SEM fractographs were used for the measurements, it should be kept in mind that the actual projection of the cleavage facets to the plane of the image was measured. Namely, the inclination of the facets from the plane normal to the direction of the tensile stress was not taken into account. Previous investigations have shown that the angles of the cleavage facets to the plane normal to the direction of maximal principal tensile stress applied do not exceed 15° [33]. It follows that the effective diameters of the first cleavage facets for V and TiV steel are not significantly different. The distribution of the max principal stress and plastic strain along the distances from the notch root for all 4PB specimens, calculated by FEM, is presented in the diagrams in Figure 10.

While the values of critical plastic strain, ε_pc_, are much lower for the TiV steel (0.0116–0.0264) than for the V steel (0.0299–0.0648), the values of the critical stress σ_F_* are somewhat similar (around 1500 MPa). Peak values of the maximum stress, σ_1max_, for the V steel samples are in the range 1722–1876 MPa, while for the TiV steel samples they are considerably lower, in the range 1617–1779 MPa. Overall, for the V steel both the values of critical stress σ_F_* and the values critical plastic strain ε_pc_ are higher than for the TiV steel. Additionally, the position of the stress peak value X_0_ is further away for V steel (0.2550–0.3098 mm), than for the TiV steel samples (0.1815–0.2392 mm). The difference in magnitude of plastic strain in front of the notch root at the fracture between the two steels, presented in Figure 10, can be explained by the difference in volume fraction of cementite and pearlite that is directly correlated with the volume fraction of AF. Considering the finding that coarse spherical second phase particles were not responsible for the cleavage fracture initiation in the structure dominated by acicular ferrite in V and TiV microalloyed medium carbon steels, other possible mechanisms are considered, such as Smith’s mechanism of cleavage fracture initiation by fracture of cementite plates at the ferrite grain boundaries [26,35] or the Miller–Smith mechanism of pearlite nodule fracture [37,46].

Assuming Smith’s mechanism of cleavage fracture initiation, stress for the cementite plate fracture can be calculated using Griffith’s equation for through-thickness microcrack:

(4) $$\sigma_{F}^{*}=\sqrt{\frac{4E\gamma }{\pi \left(1-\nu \right)C}}$$

Taking the values of effective surface energy, γ, of 9 Jm^−2^ [47], modulus of elasticity, E, of 189 GPa and Poisson’s coefficient, ν, of 0.301 for cementite [48], and taking the average maximal thickness of cementite plates measured in this work of 0.798 µm and 0.966 µm, the calculation renders stress values of 1970 MPa and 1791 MPa, for V and TiV steel respectively. When plotted in the stress distribution graphs in Figure 10a,b, it is clear that a fracture of the cementite plates could be possible in the narrow zone around the peak stress. Additionally, the position and orientation of the cementite plates in AF is related to the irregular distribution of the sheaves of ferrite plates. In that manner, most of the cementite plates are not favorably oriented in respect to the normal tensile stress. Therefore, cleavage fracture initiation in the zone of peak stress, at low plastic strains, by fracture of thick cementite plates placed between acicular ferrite plate sheaves, seems impossible. On the other hand, stresses near the notch root are not sufficient for fracture of the cementite plates (Figure 10). Nevertheless, in all samples the cleavage fracture was initiated from the sites near the notch root. Therefore, it can be concluded that Smith’s mechanism of cleavage fracture initiation at such short distances from the notch root was not possible.

Furthermore, the point that peak values of the normal tensile stress, σ_11_, located away from the notch root were not sufficient for fracture of coarse TiN particles, which is a common mechanism of the cleavage fracture initiation in medium carbon microalloyed steels [29]. Taking the values of the peak stresses in the range from approximately 1722 to 1876 MPa for V steel and 1617 to 1779 MPa for TiV steel, and the effective surface energy for fracture of TiN particles of 7 Jm^−2^ [34,47], the minimum size of spherical TiN particles that could fracture can be calculated using Griffith’s equation for spherical particles [47,48,49,50]:

(5) $$\sigma_{p/m}=\sqrt{\frac{\pi E\gamma_{p/m}}{\left(1-\nu^{2}\right)d}}$$

The calculation renders diameters of the particles in the range from 1.3–1.6 μm for V steel, and 1.5–1.8 μm for TiV steel, meaning that larger particles could be eligible as a cleavage fracture nucleation site. In most cases in literature, TiN particles of 2–6 μm in diameter were responsible for the cleavage fracture initiation in medium carbon microalloyed steels with ferrite-pearlite, bainite, or martensite structure [18,27,33,34,35,36,37,44,45]. It could mean that TiN particle fracture stress is lower than the stress for the microcrack propagation into the matrix. In that case, propagation of the microcrack formed at the particle cannot propagate into a matrix and eventually blunts. However, in this work no broken second phase particles or blunted microcracks were found in the zone of peak stress. It remains unclear why TiN particles remain inactive as a cleavage fracture initiation sites even in the zone of peak stresses at the distance from the notch root. However, it could be assumed that acicular ferrite affects cleavage fracture initiation by TiN particles fracture in certain manner.

Taking into account that all sites of cleavage fracture initiation were placed in the area of high plastic strains, in the vicinity of the notch roots of the 4PB specimens, and bearing in mind the previous analysis, it follows that initial microcracks were formed by damage accumulation by plastic deformation in pearlite, a mechanism referred to as Miller–Smith’s, characteristic of high pearlitic steels [48]. When the lamellae in pearlite are aligned at angles close to 45° relative to the direction of applied tension, dislocations move freely in the ferrite matrix without encountering ferrite/cementite interfaces, allowing significant plastic deformation without straining the cementite phase. However, when aligned parallel or perpendicular to the tensile direction, the lamellar cementite must accommodate the plastic deformation of the ductile ferrite matrix, and the cementite plates break [25]. Formed microcracks in cementite readily propagate into neighboring ferrite lamellae due to the crystallographic relationship in pearlite. By tearing the pearlite nodules under the plastic strains, relatively large microcracks are formed, which can easily propagate at low stress in the vicinity of the notch root, compared to microcracks created by the fracture of second-phase particles.

Further, it follows that the stresses at which microcracks are formed are equal to or greater than the stress needed for the microcrack’s propagation into the matrix. Moreover, considering the microstructures of the examined steels, it can be elucidated that the crack propagates into the AF that surrounds the pearlite placed right next to the grain boundary ferrite (Figure 4, Figure 5 and Figure 6). Therefore, a few important conclusions can be drawn. Firstly, the stress for crack initiation by second-phase particle fracture was not attained. Therefore, it could be concluded that it is higher than the recorded stress at which the initial crack propagated. Considering that the cleavage fracture is a three-stage process: (1) microcrack formation, (2) microcrack propagation through the boundary with the matrix, and (3) further crack propagation through the high-angle boundaries in the matrix [51], it follows that in this case, where cleavage was initiated by tearing of the perlite, the critical stage of the cleavage process is microcrack formation. Once formed, it propagates at low stress in a dynamic manner without stopping at high angle boundaries, and thus:

σ_p_ > σ_p/m_ > σ_m/m_

where σ_p_ is the stress for microcrack formation by second phase particle fracture, σ_p/m_ is the stress for the initial microcrack propagation particle/matrix boundary, and σ_m/m_ is the stress for the crack propagation through the high-angle boundaries in the matrix.

Secondly, the critical fracture stress in this case is equal to the stress for propagation through AF and thus depends on its structure in the sense of thickness and mutual crystallographic misorientation of the ferrite plates. It is generally accepted that the influence of AF structure is related to the process of crack propagation through the structure with a high density of high-angle boundaries, forcing it to frequently change propagation direction, which requires additional fracture energy [52]. However, current results suggest that the AF structure hinders fracture initiation at the coarse second-phase particles in a certain manner. It can be assumed that second-phase particles, primarily individual cementite plates separated by interlocked ferrite plates, are less favorably oriented toward acting tensile stress. Moreover, it should also be kept in mind that specific deformation behavior is due to the higher dislocation density inherent in AF and B structures [28,53]. Namely, it is characterized by relatively low yield stress due to the effect of gradual yielding, leading to plastic yielding at the notch, thus lowering the overall stress level in the 4PB specimen. It could be speculated that in the same manner, a part of the energy is spent on dislocation glide in the AF matrix around the particles.

**Figure 11:**
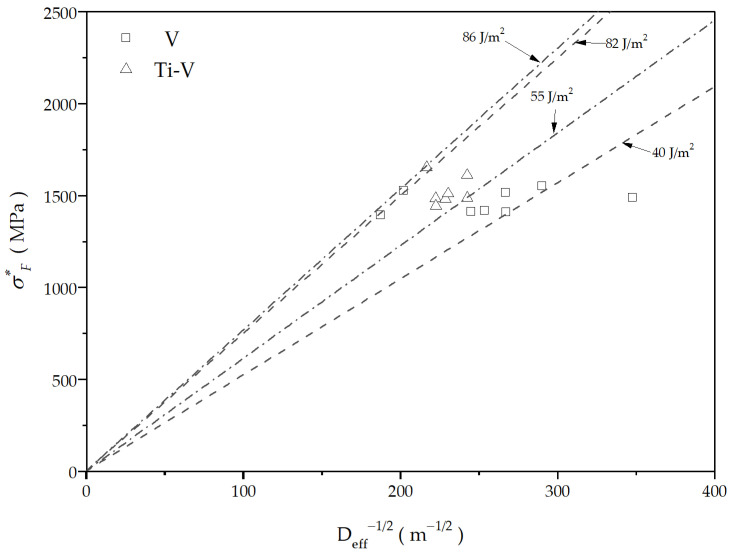
Values of the local critical fracture stress displayed against the reciprocal square root of the effective diameter of potential first cleavage fracture facet.

The values of critical fracture stresses for both steels examined are plotted against reciprocal square root values of effective cleavage facet diameters in the graph shown in Figure 11. Lines have been drawn below the lowest and highest experimental points for each steel. In accordance with Griffith’s Equation (5), the slope of the line represents the effective surface energy, γ.

The values of effective surface energy for the V steel ranges from 40 Jm^−2^ to 86 Jm^−2^, while in the case of TiV steel it is noticeably similar, ranging from 55 Jm^−2^ to 82 Jm^−2^. These values are comparable to those found in the literature for medium carbon microalloyed steels with ferrite-pearlite or bainite structures [27,41,44,53]. The observed difference in effective surface energy can be related to the effect of AF in the structure of the steels. On one hand, there is considerably higher volume fraction of AF in TiV steel, but on the other hand, it is the pearlite that is involved in the fracture initiation. While the AF’s fine interlocking structure may contribute to the increase of the local fracture stress, low-ductility coarse ferrite-pearlite aggregates with the same crystallographic orientation reduce the critical cleavage fracture stress as a suitable site for cleavage fracture initiation. This is especially noticeable in V steel, where more pearlite nodules and proeutectoid ferrite grains provide more potential sites for the cleavage fracture initiation at lower stress levels close to the notch root. Therefore, it can be concluded that the critical cleavage fracture stress in V and TiV microalloyed medium carbon steels is determined by the stress for propagation of the crack through the matrix, consisting of AF, pearlite and proeutectoid ferrite. At the same time, due to the specific nature of the cleavage fracture initiation in this case, local critical fracture stress cannot be directly related to the nominal fracture stress, σ_F_ (Table 2) as in cases with medium carbon microalloyed steels with classical ferrite-pearlite, bainite, or martensite structures [29,44], i.e., σ_F_* > σ_F_.

## 4. Conclusions

1. The fracture of pearlite nodules during plastic deformation at the notch root of the four-point bending specimen initiates cleavage fracture of air-cooled medium-carbon microalloyed steels with primarily acicular ferrite microstructure. The cleavage fracture initiation procedure in this example did not involve the fracturing of coarse second-phase particles or thick cementite plates within AF;
2. Peak stress in front of the notch root is insufficient for the cleavage fracture initiation by fracture of coarse TiN particles in the microstructure consisting primarily of acicular ferrite due to its fine-grained structure with a high density of high-angle boundaries, as compared to the ferritic–pearlitic structures;
3. The average maximal thickness of cementite plates placed at AF plate boundaries was 0.7984 µm and 0.9656 µm, for V and TiV steel, respectively. The corresponding stress values required for their fracture according to Griffith’s equation are 1970 MPa and 1791 MPa, respectively, which is well below the stress levels at the cleavage initiation sites near the notch root. Therefore, the fracture of coarse cementite plates is not responsible for the cleavage fracture initiation;
4. Estimated values of the effective surface energy for the V steel ranges from 40 Jm^−2^ to 86 Jm^−2^, and for the TiV steel from 55 Jm^−2^ to 82 Jm^−2^. These values correspond to the energy required for cleavage crack propagation through the grain boundaries, and therefore reflect the effect of the interlocking structure of acicular ferrite;
5. The critical cleavage fracture stress in V and TiV microalloyed medium carbon steels with predominantly AF structures corresponds to the stress for crack propagation through the matrix;
6. The critical stage in the cleavage fracture process of continuously cooled V and TiV microalloyed medium carbon steels with predominantly AF structures, together with proeutectoid ferrite and pearlite, is the initial microcrack formation.

## Figures and Tables

**Table 1:** Chemical composition of steels/wt %.

Steel	C	Si	Mn	P	S	Cr	Ni	Mo	V	Ti	Al	Nb	N
V	0.256	0.416	1.451	0.0113	0.0112	0.201	0.149	0.023	0.099	0.002	0.038	0.002	0.0229
TiV	0.309	0.485	1.531	0.0077	0.0101	0.265	0.200	0.041	0.123	0.011	0.017	0.003	0.0228

**Table 2:** Force parameters used for cleavage fracture FEA modelling.

Sample Series	σ_f_ (MPa)	Experiment Load F_max_ (N)	Modeled Displacement U2 (mm)	Sample Series	σ_f_ (MPa)	Experiment Load F_max_ (N)	Modeled Displacement U2 (mm)
V-01	11,927	28,425.84	−0.2234	TiV-01	758.7	18,152.24	−0.1382
V-02	1267.8	30,319.96	−0.239	TiV-02	934.1	22,335.16	−0.1699
V-03	1049.0	25,083.64	−0.1952	TiV-03	904.3	21,659.12	−0.1647
V-04	1152.5	27,652.4	−0.2167	TiV-04	950.3	22,755.56	−0.1732

**Table 3:** Statistical analysis of the area fraction for all three phases executed for both steels.

Steel	Phase	Mean Area Fraction (%)	Min Area Fraction (%)	Max Area Fraction (%)	Median Area Fraction (%)	Std. Dev. Area Fraction (%)
V	Ferrite	41.83	29.24	63.85	41.67	5.26
	Pearlite	54.16	28.51	69.14	54.29	6.44
	Cementite	3.78	1.06	9.21	3.66	1.34
TiV	Ferrite	69.93	62.15	74.76	70.28	2.27
	Pearlite	10.56	3.36	23.32	9.81	3.66
	Cementite	18.30	11.95	22.38	18.43	1.81

**Table 4:** Statistical analysis of cementite phase local thickness done for both steels.

Sample Series	Measurement	Mean Local Thickness (μm)	Minimum Local Thickness (μm)	Maximum Local Thickness (μm)	Median Local Thickness (μm)	Std. Dev. Local Thickness (μm)
V	Average	0.2644	0.1176	0.7984	0.2476	0.1361
	Min	0.2464	0.1176	0.7435	0.2351	0.1289
	Max	0.2829	0.1176	0.9478	0.2629	0.1423
TiV	Average	0.3738	0.1176	0.9656	0.3741	0.1362
	Min	0.3369	0.1176	0.8477	0.3527	0.1296
	Max	0.4124	0.1176	1.1090	0.4239	0.1456

**Table 5:** Critical parameters for cleavage fracture initiation.

Sample Series	X_0_ (μm)	σ_1max_ (MPa)	σ_F_* (MPa)	ε_pc_	D_max_ × D_min_ (μm)	D_eff_ (μm)	γ (Jm^−2^)
V-01	41.1	1822	1527	0.04383	36.8 × 15.5	24.5	84.0
	49.7		1552	0.04107	12.8 × 17.3	11.9	42.0
V-02	17.4	1876	1419	0.06200	23.7 × 9.8	15.6	46.0
	16.0		1413	0.06287	21.0 × 11.3	16.7	48.9
	12.2		1396	0.06483	29.3 × 24.1	28.5	81.5
V-03	19.4	1722	1411	0.03969	41.0 × 8.1	14.0	41.0
	54.0		1489	0.02992	8.6 × 6.8	8.3	26.9
V-04	40.8	1798	1516	0.04732	34.7 × 8.2	14.0	47.4
Ti–V-01	78.7	1617	1485	0.01164	20.4 × 18.3	20.2	65.4
Ti–V-02	58.6	1765	1488	0.02183	18.8 × 12.7	17.0	55.2
	55.8		1480	0.02234	22.3 × 13.6	19.1	61.5
Ti–V-03	112.1	1744	1612	0.01351	17.2 × 15.3	17.0	64.9
	130.7		1654	0.01165	22.1 × 17.6	21.3	85.5
Ti–V-04	40.8	1779	1443	0.02639	35.4 × 12.3	20.2	61.7
	65		1511	0.02164	29.1 × 11.8	18.8	63.1

## Data Availability

The data presented in this study are available on request from the corresponding author. The data are not publicly available.
